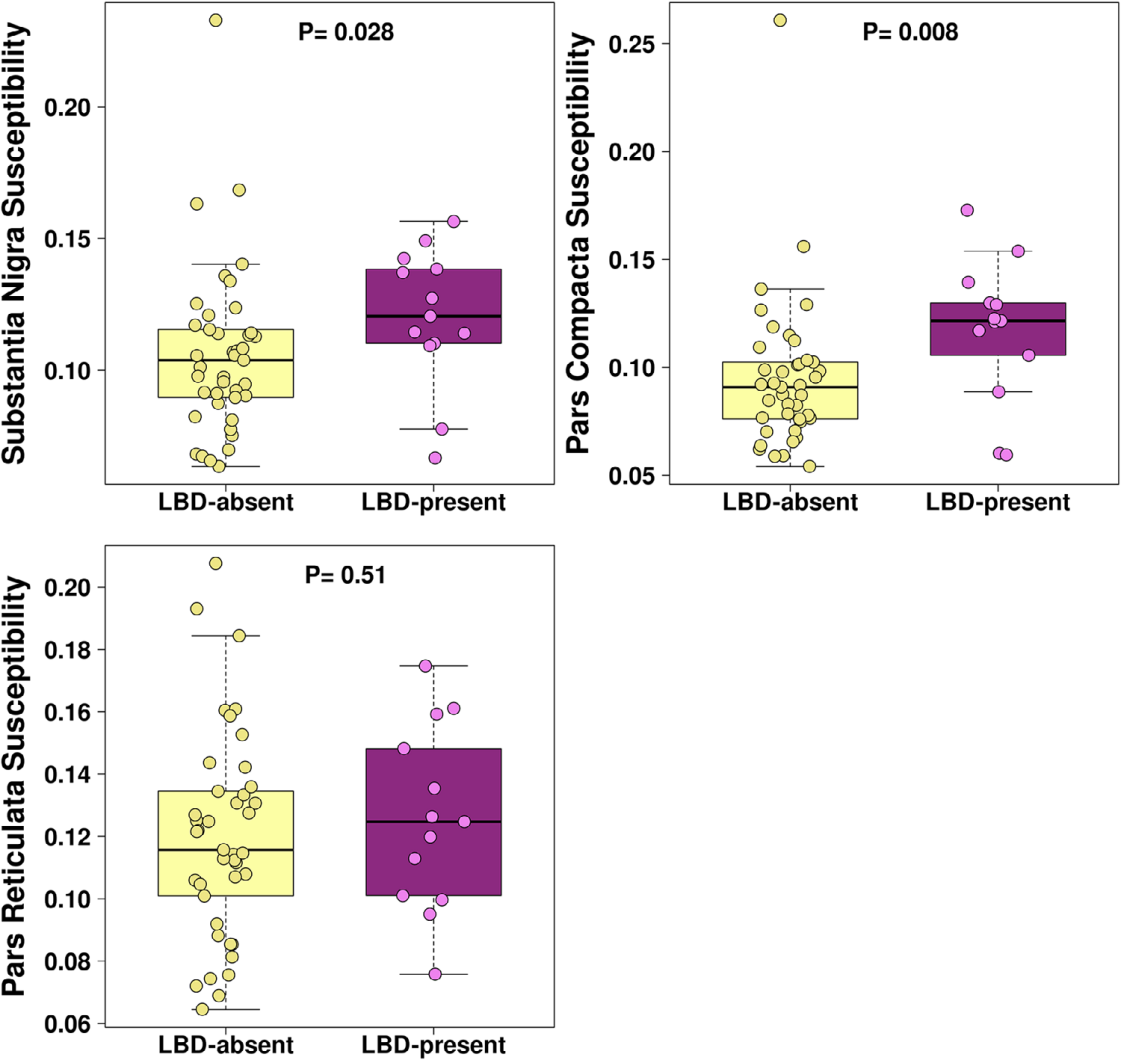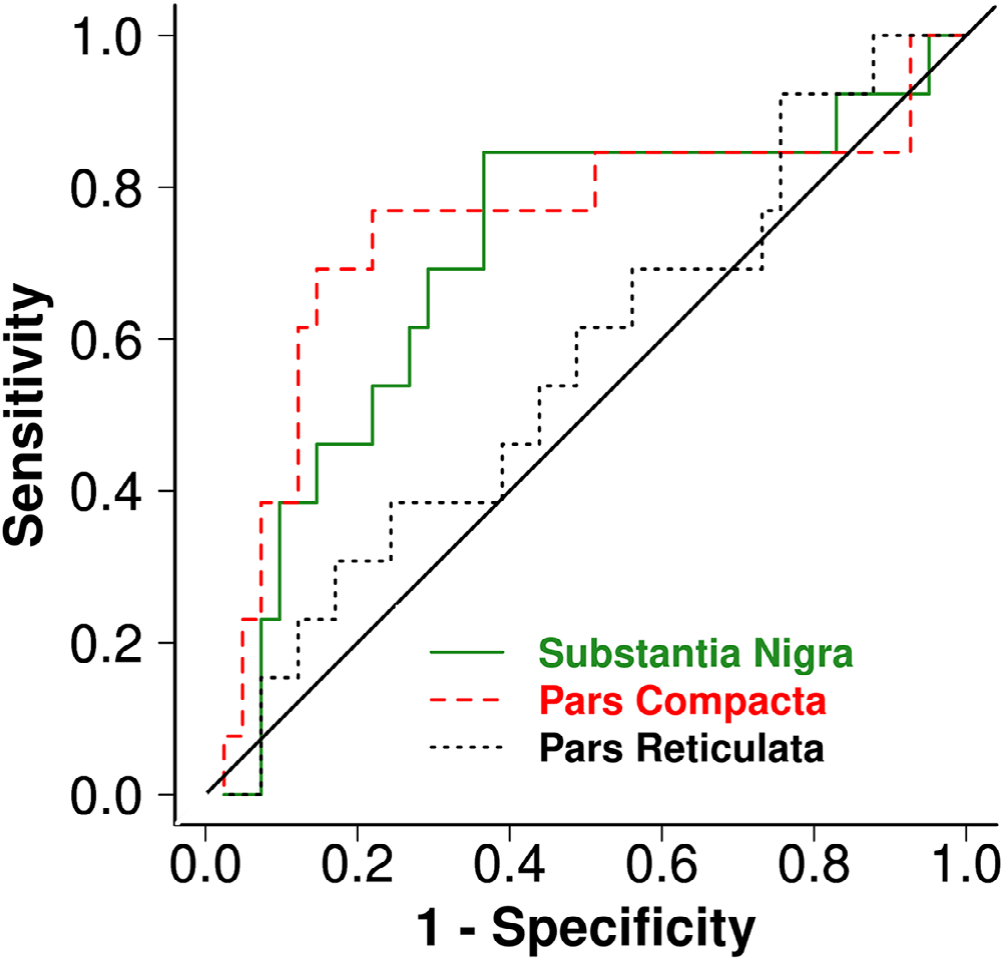# Substantia nigra iron deposition in Lewy body disease: an MRI quantitative susceptibility mapping and neuropathology study

**DOI:** 10.1002/alz.091491

**Published:** 2025-01-09

**Authors:** Patricia Diaz‐Galvan, Scott A. Przybelski, Timothy G. Lesnick, Aivi T. Nguyen, Melissa E. Murray, Ross R. Reichard, Dennis W. Dickson, Daisuke Ono, Christopher G. Schwarz, Matthew L. Senjem, Jeffrey L. Gunter, Val J. Lowe, Leah K. Forsberg, Julie A. Fields, Rodolfo Savica, Jonathan Graff‐Radford, Vijay K. Ramanan, David T. Jones, Hugo Botha, Erik K St. Louis, David S. Knopman, Gregory S Day, Neill R Graff‐Radford, Ronald C. Petersen, Clifford R. Jack, Tanis J Ferman, Brad F. Boeve, Kejal Kantarci

**Affiliations:** ^1^ Department of Radiology, Mayo Clinic, Rochester, MN USA; ^2^ Department of Quantitative Health Sciences, Mayo Clinic, Rochester, MN USA; ^3^ Mayo Clinic, Quantitative Health Sciences, Rochester, MN USA; ^4^ Mayo Clinic, Rochester, MN USA; ^5^ Mayo Clinic, Jacksonville, FL USA; ^6^ Department of Laboratory Medicine and Pathology, Mayo Clinic, Rochester, MN USA; ^7^ Department of Neuroscience, Mayo Clinic, Jacksonville, FL USA; ^8^ Department of Neurology, Mayo Clinic, Rochester, MN USA; ^9^ Department of Psychiatry and Psychology, Mayo Clinic, Rochester, MN USA; ^10^ Department of Neurology, Mayo Clinic, Jacksonville, FL USA; ^11^ Department of Psychiatry and Psychology, Mayo Clinic, Jacksonville, FL USA

## Abstract

**Background:**

Parkinson’s disease and dementia with Lewy bodies are characterized by abnormal iron deposition in the substantia nigra, which can be measured with quantitative susceptibility mapping (QSM) on MRI. However, neuropathologic validation of the increased QSM in the substantia nigra associated with Lewy body disease (LBD) is lacking. We compared substantia nigra QSM on antemortem MRI between patients with and without Lewy‐related pathology which was confirmed by autopsy in all cases.

**Method:**

We studied cases with antemortem QSM who underwent autopsy from the Mayo Clinic Alzheimer’s Disease Research Center and Mayo Clinic Study of Aging (n=54). Immunohistochemistry was performed with rabbit NACP antibody against the amino acids 98‐115 of the α‐synuclein protein to detect Lewy‐related pathology. After neuropathologic evaluation, cases were classified as LBD‐present (n=13) if they had Lewy‐related pathology and LBD‐absent (n=42) if they did not have Lewy‐related pathology. Alzheimer’s disease co‐pathology was present in 9 of the LBD‐present cohort (70%). QSM was calculated for the whole substantia nigra and the two sub‐regions: pars compacta and pars reticulata. Non‐parametric Wilcoxon rank‐sum test was used to compare substantia nigra QSM values between LBD‐present and LBD‐absent cases. Area under the curve (AUC) analyses tested the accuracy of QSM to distinguish the two groups.

**Results:**

LBD‐present group had higher QSM values in the substantia nigra than LBD‐absent group. Furthermore, sub‐regional analyses showed that the LBD‐present group had higher QSM in the substantia nigra pars compacta (p=0.008) but not in the pars reticulata than the LBD‐absent group (Figure 1). QSM of the substantia nigra pars compacta distinguished LBD‐present and LBD‐absent cases with good accuracy (AUC=0.74; p=0.006; Figure 2).

**Conclusion:**

This study provides neuropathologic insight into the relevance of QSM of substantia nigra pars compacta as an in‐vivo biomarker of autopsy‐confirmed LBD. These findings indicate that imaging evidence of abnormal iron deposition in the substantia nigra may serve as a biomarker of Lewy‐related pathology in patients with primary LBD (i.e., Parkinson disease, dementia with Lewy bodies) and patients with potential LBD co‐pathology (e.g., patients with Alzheimer disease).